# Immunobiotic *Lactobacillus jensenii* as immune-health promoting factor to improve growth performance and productivity in post-weaning pigs

**DOI:** 10.1186/1471-2172-15-24

**Published:** 2014-06-19

**Authors:** Yoshihito Suda, Julio Villena, Yu Takahashi, Shoichi Hosoya, Yohsuke Tomosada, Kohichiro Tsukida, Tomoyuki Shimazu, Hisashi Aso, Masanori Tohno, Mitsuharu Ishida, Seiya Makino, Shuji Ikegami, Haruki Kitazawa

**Affiliations:** 1 Department of Food, Agriculture and Environment, Miyagi University, Sendai, Japan; 2 Food and Feed Immunology Group, Laboratory of Animal Products Chemistry, Graduate School of Agricultural Science Tohoku University, 981-8555 Sendai, Japan; 3 Laboratory of Animal Breading and Genetics, Graduate School of Agricultural Science, Tohoku University, Sendai, Japan; 4 Cell Biology Laboratory, Graduate School of Agricultural Science, Tohoku University, Sendai, Japan; 5 National Institute of Livestock and Grassland Science, Nasushiobara, Japan; 6 Division of Research and Development, Meiji Co., Ltd, Kanagawa, Japan; 7 Laboratory of Immunobiotechnology, CERELA-CONICET, Tucuman, Argentina

**Keywords:** Immunobiotics, Immune performance, Productivity, Piglets, *Lactobacillus jensenii* TL2937, TLR4, TLRs negative regulators

## Abstract

**Background:**

Immunoregulatory probiotics (immunobiotics) have been proposed to improve piglets’ immune system to avoid intestinal infections and reduce unproductive inflammation after weaning. Previously, it was demonstrated that *Lactobacillus jensenii* TL2937 (LjTL2937) attenuated the inflammatory response triggered by activation of Toll-like receptor 4 (TLR-4) in porcine intestinal epithelial (PIE) cells and antigen presenting cells (APCs) from porcine Peyer’s patches (PP).

**Objective:**

In view of the critical importance of PIE-APCs interactions in the regulation of intestinal immune responses, we aimed to examine the effect of LjTL2937 on activation patterns of APCs from swine PPs in co-cultures with PIE cells. In addition, we investigated whether LjTL2937 was able to beneficially modulate intestinal immunity of piglets after weaning to improve immune-health status.

**Results:**

Stimulation of PIE-APCs co-cultures with LjTL2937 increased the expression of MHC-II, CD80/86, IL-10, and Bcl-3 in CD172a^+^CD11R1^-^ and CD172a^+^CD11R1^high^ APCs. In addition, the TL2937 strain caused the upregulation of three negative regulators of TLR4 in PIE cells: MKP-1, Bcl-3 and A20. These changes significantly reduced the inflammatory response triggered by TLR4 activation in PIE-APCs co-cultures. The in vivo experiments using castrated male piglets (crossbreeding (LWD) with Landrace (L), Large Yorkshire (W) and Duroc (D))of 3 weeks of age demonstrated that feeding with LjTL2937 significantly reduced blood complement activity and C reactive protein concentrations while no changes were observed in blood leukocytes, ratio of granulocytes to lymphocyte numbers, macrophages’ activity and antibody levels. In addition, treatment with LjTL2937 significantly improved growth performance and productivity, and increased carcass quality.

**Conclusions:**

We demonstrated that the use of immunobiotics strains like LjTL2937, as supplemental additives for piglets feedings, could be used as a strategy to maintain and improve intestinal homeostasis; that is important for the development of the pig and for health and performance throughout the productive life of the animal.

## Background

Intensification of the pig industry has brought increased risks of both clinical and sub-clinical enteric disease. The neonatal pig is immunologically incompetent until about 4 weeks of age. Thus the period from birth through weaning represents a critical time for pigs [1]. In piglets, weaning involves multiple changes; they switch from a liquid to a solid diet, they are taken away from their mothers, and they are moved to unfamiliar buildings where they may be exposed to new environmental antigens. These changes trigger transit inflammatory responses in the gut that can contribute to anatomical and functional intestinal disorders [2-4]. In fact, weaning and transport stress enhance the vulnerability to colonization by pathogenic bacteria. Piglets are vulnerable to potentially harmful microorganisms such as enterotoxigenic *Escherichia coli* (ETEC), *Salmonella* spp. and *Clostridium perfringens *[5]. Therefore, controlling early intestinal inflammation is a major challenge in managing post-weaning gut disorders in piglets. Several attempts have been made to alleviate weaning stress and the related immunological disorders. Antibiotics have been applied widely to prevent and treat gastrointestinal infection in piglets, however the promiscuous use of antibiotics resulted in the spread of resistant bacteria [6,7].

Recently, probiotic microorganisms have been proposed as an alternative to avoid non-protective inflammation and to improve resistance against intestinal infections in piglets [8-10]. Probiotic lactic acid bacteria (LAB) able to modulate the immune system (immunobiotics) are known to play a beneficial role in the prevention and therapy of a variety of intestinal inflammatory disorders [11,12]. In this regard, we demonstrated that *Lactobacillus jensenii* TL2937 attenuates the expression of proinflammatory cytokines and chemokines triggered by ETEC or lipopolysaccharide (LPS) in a porcine intestinal epitheliocyte (PIE) cell line [9]. *L. jensenii* TL2937 attenuates proinflammatory responses in PIE cells by downregulating Toll-like receptor (TLR)-4-dependent nuclear factor κB (NF-κB) and mitogen- activated protein kinase (MAPK) activation. Furthermore, we demonstrated that *L. jensenii* TL2937 stimulation of PIE cells results in upregulation of three negative regulators of TLRs, the ubiquitin-editing enzyme A20, B-cell lymphoma 3-encoded protein (Bcl-3), and mitogen-activated protein kinase 1 (MPK-1), and that these effects are partially dependent on the activation of TLR2 [9]. More recently, we evaluated the effect of the TL2937 strain on antigen presenting cells (APCs) from porcine Peyer’s patches (PPs) and we found that direct exposure of porcine APCs to *L. jensenii* TL2937 in the absence of inflammatory signals activated CD172a^+^ APCs and caused them to become phenotypically and functionally mature and to display tolerogenic properties [10]. We also demonstrated that pretreatment of APCs with *L. jensenii* TL2937 resulted in differential modulation of the production of pro- and anti-inflammatory cytokines in response to ETEC or LPS challenge [10]. The immunomodulatory effect of strain TL2937 was not related to a downregulation of TLR4 but was related to an upregulation of the expression of three negative regulators of TLRs: single immunoglobulin IL-1-related receptor (SIGIRR), A20, and interleukin-1 receptor-associated kinase M (IRAK-M).

Our results in monocultures of intestinal epithelial cells (IECs) or APCs clearly showed the anti-inflammatory potential of *L. jensenii* TL2937. However, these in vitro models are simplified and may neglect the effect of cell–cell interactions in a complex organic microenvironment, which completely changes the resulting response. IECs express a broad range of factors that may influence intestinal APCs and lymphocytes [13,14]. In the steady state, IECs create a tolerogenic environment that favors the promotion and development of tolerogenic APCs and CD4^+^CD25^+^Foxp3^+^ Treg cells [14,15]. However, in the presence of pathogenic bacteria, IECs function as APCs to different subsets of T cells [16] and, moreover, through the secretion of interleukin (IL)-1, IL-6, IL-8, IL-18 and tumor necrosis factor (TNF), play a role in the activation of innate immune response [17]. Thus, together with local immune cells, it is the intestinal epithelium that governs the induction of oral tolerance or inflammation. Then, in view of the critical importance of IECs-APCs interaction on the regulation of intestinal immune responses, the aim of the present study was to examine the effect of *L. jensenii* TL2937 on activation patterns of APCs from swine PPs in co-cultures with PIE cells. Therefore, we evaluated the functional consequences of indirect exposure of APCs to *L. jensenii* TL2937 under non-inflammatory and inflammatory conditions. In addition, this study aimed to investigate whether the in vitro effects of *L. jensenii* TL2937 reported in previously published works [9,10] and extended here were able to beneficially modulate intestinal immunity of piglets after weaning to improve immune-health status and productivity.

## Methods

### In vivo and ex vivo experiments

This study was carried out in strict accordance with the recommendations in the Guide for the Care and Use of Laboratory Animals of the Guidelines for Animal Experimentation of Miyagi University, Sendai, Japan. The present study was approved by the Laboratory Health and Safety Committee of Miyagi University with a permitted No. H20-10 and all efforts were made to minimize suffering.

### Microorganisms

Enterotoxigenic *Escherichia coli* (ETEC) strain 987 was kindly provided by Dr. M. Nakazawa at the National Institute of Animal Health (Tsukuba, Japan) [9,10]. ETEC cells were grown in blood agar for 24 hours at 37°C and then transferred to tryptic soy broth (TSB; Becton Dickinson and Company, San Jose, CA) for 5 days at 37°C without shaking. The 5 days period is necessary for the cells to form a pellicle containing the piliated phase. Then, ETEC cells were collected from the pellicle and transferred to TSB and cells were grown for 24 h at 37°C with shaking. After overnight incubation, bacteria from subcultures were centrifuged at 5,000 g for 10 min at 4°C, washed with phosphate-buffered saline (PBS), and heat killed (100°C, 30 min). Each culture of the two Lactobacillus strains (*L. jensenii* TL2937 and *L. plantarum* TL2766) was grown in a sterile medium composed by 10% whey powder (w/v) hydrolyzed with 0.1% (w/v) protease A (Amano Enzyme Inc., Nagoya, Japan) for 3 h at 50°C then added with 0.5% (w/v) yeast extract. Growth was performed for for 16 h at 37°C, washed with PBS, and heat inactivated (56°C, 30 min). These bacterial samples were resuspended in Dulbecco’s modified Eagle medium (DMEM), enumerated using a microscope and a Petroff-Hausser counting chamber, and stored at -80°C until use [9,10].

### Isolation of immune cells from swine Peyer’s patches

Suspensions of porcine PP immunocompetent cells were prepared from the ilea of adult swine as previously described [10,18,19]. All procedures were conducted in accordance with the Guidelines for Animal Experimentation of Tohoku University, Sendai, Japan. Briefly, PPs were cut into small fragments; the fragments were then gently pressed through a nylon mesh and washed three times in complete RPMI 1640 medium (Sigma, St. Louis, MO) supplemented with 10% fetal calf serum (FCS; Sigma). Residual erythrocytes were lysed by resuspension in hypotonic salt solution (0.2% NaCl). Next, harvested PP cells were subjected to hypertonic rescue in an equal volume of 1.5% NaCl. Finally, immune cells were fractionated using Lympholyte-mammal (Cedarlane, Hornby, Ontario, Canada) density gradient centrifugation, and the isolated immune cells were suspended in complete DMEM (Invitrogen, Tokyo, Japan) supplemented with 10% FCS (Sigma), 50 g/ml penicillin-streptomycin, and 50 g/ml gentamicin (Nacalai Tesque, Kyoto, Japan).

### Isolation of adherent population from swine Peyer’s patches

We isolated APCs (DCs and macrophages) from porcine PP tissue samples by culturing the mononuclear cells from these samples on glass plates and selecting the adherent cells as described previously [10]. Briefly, after mononuclear cells were isolated from swine PP samples as described above, cell suspensions were adjusted to a concentration of 5 × 10^7^ cells/ml. Cell suspensions (1 ml/well) were placed into 2-well glass plates (Iwaki, Tokyo, Japan) and incubated for 2 h at 37°C (5% CO_2_ atmosphere) to allow cells to adhere to the glass surface. Subsequently, these glass plates were washed gently with complete RPMI 1640 medium (Sigma) to remove non-adherent cells. Remained cells are referred to as adherent cells.

### PIE cells

PIE cells, which are non-transformed intestinal cultured cells originally derived from intestinal epithelia isolated from an unsuckled neonatal swine [9,10], were maintained in Dulbecco’s modified Eagle’s medium (DMEM) (Invitrogen Corporation, Carlsbad, CA) supplemented with 10% fetal calf serum (FCS), 100 mg/ml penicillin, and 100 U/ml streptomycin at 37°C in an atmosphere of 5% CO2. PIE cells grow rapidly and are well adapted to culture conditions even without transformation or immortalization. However, the proliferative ability of PIE cells diminishes after 50 passages in culture. Therefore, we used PIE cells only between the 20^th^ and 40^th^ passages in these experiments.

### PIE and adherent cells co-culture system

In the Transwell culture system, PIE cells were seeded in the apical compartment at a concentration of 1.5 × 10^5^ cells/well in 12-well tissue culture plates (Transwell-COL [PTFE]; pore size, 0.2 mm), and adherent cells from porcine PPs were seeded in the basolateral compartment at a concentration of 2 × 10^7^ cells/well. For the evaluation of lactobacilli immunomodulatory activities in the PIE-APC cell co-culture system, PIE cells in the apical compartment were stimulated with lactobacillus strains (5 × 10^7^ cells/ml) for 48 h. For the evaluation of lactobacilli anti-inflammatory, PIE cells in the apical compartment were stimulated with lactobacillus strains (5 × 10^7^ cells/ml) for 48 h, washed twice with PBS and stimulated with ETEC (5 × 10^7^ cells/ml) for 12 h. Studies of protein expression of different cytokines were performed using the flow cytometric analysis described below. In addition, the expression of specific mRNAs in PIE and APC cells was studied by real-time PCR as described below.

### Flow cytometric analysis

Previous flow cytometric analysis of porcine PP adherent cells showed that it was possible to identify the three populations of APCs detected in mononuclear cells isolated from fresh PPs: CD172a^+^CD11R1^-^, CD172a^+^CD11R1^high^, and CD172a^-^CD11R1^low^ adherent cells [10]. This method of APC isolation did not completely eliminate CD172a^-^CD11R1^-^ cells (which include T and B cells) from the cultures; however, it did allow us to harvest samples with a high proportion of APCs. Then, flow cytometry was used to assess expression of MHC-II and several cytokine proteins in CD172a^+^CD11R1^-^, CD172a^+^CD11R1^high^, and CD172a^-^CD11R1^low^ adherent cells from PPs. Cells were labeled with primary antibodies: anti-porcine CD172a-PE SWC3 IgG1 (Southern Biotech) (1/50 dilution), anti-porcine CD11R1-un- labeled IgG1 (AbD Serotec) (1/50 dilution), anti-porcine MHC-II-unlabeled IgG2a (VMRD) (1/100 dilution), anti-porcine gamma interferon (IFN-γ)-unlabeled IgG2b (R&D Systems, Minneapolis, MN) (1/20 dilution), anti-porcine interleukin-10 (IL-10)-unlabeled IgG2b (R&D Systems) (1/20 dilution), anti-porcine IL-1β/IL-1 F2-unlabeled IgG1 (R&D Systems) (1/20 dilution), anti-porcine IL-6-unlabeled IgG2b (R&D Systems) (1/20 dilution), and anti-porcine transforming growth factor β2 (TGF-β2)-unlabeled IgG (R&D Systems) (1/20 dilution). The binding of unlabeled monoclonal antibodies was visualized using the following secondary antibodies: anti-mouse IgG1- peridinin chlorophyll protein (PerCP)/Cy5.5 (Bio Legend, San Diego, CA) (1/100 dilution), anti-mouse IgG2a-FITC (AbD Serotec), anti-rabbit IgG-Alexa Fluor 489 (Santa Cruz) (1/200 dilution), anti-mouse IgG2b-FITC (AbD Serotec) (1/200 dilution), and anti-mouse IgG-FITC (AbD Serotec) (1/100 dilution). In addition, expression levels of CD80/86 proteins were evaluated using a human CD152 (cytotoxic-T- lymphocyte-associated antigen 4) Ig/FITC fusion protein (Ancell, Bay- port, MN) (1/20 dilution). Cells stained with irrelevant mouse IgG-FITC, IgG2b-FITC, IgG2a-PerCP, IgG2b-PE, IgG2a-PE, or IgG1-PE antibodies (eBioscience, San Diego, CA) (1/100 dilution) were included as isotype controls. Analysis of the stained cells was performed using a FACSCalibur apparatus (BD, Franklin Lakes, NJ), which was equipped with Cell-Quest software. Data analysis was performed using FlowJo software (Tree Star, Ashland, OR).

### Quantitative expression analysis using real-time PCR

Two-step real-time quantitative PCR (qPCR) was used to characterize the expression of specific mRNAs in PIE and APC cells [9,10]. Total RNA was isolated from individual samples of porcine APCs or PIE cells using TRIzol reagent. To remove the genomic DNA, the isolated samples were treated with DNAse (PureLinkTM DNase, Cat. No. 12185–010, Invitrogen). All cDNAs were synthesized using a Quantitect reverse transcription (RT) kit (Qiagen, Tokyo, Japan) according to the manufacturer’s recommendations. Real-time quantitative PCR was carried out using a 7300 real-time PCR system (Applied Biosystems, Warrington, United Kingdom) and Platinum SYBR green qPCR SuperMix UDG with carboxy-X-rhodamine (Invitrogen). The primers used for the analysis of IL-1β, IL-6, TNF-α, IFN-γ, TGF-β and IL-10 were described previously [9,10]. The primers used to assess expression of six negative regulators of TLR signaling (single immunoglobulin IL-1-related receptor [SIGIRR], Toll-interacting protein [Tollip], interleukin-1 receptor-associated kinase M [IRAK-M], A20, Bcl-3, and MKP-1 are described by Shimazu et al. [9]. PCR cycling conditions were 2 min at 50°C, followed by 2 min at 95°C and then 40 cycles of 15 s at 95°C, 30 s at 60°C, and 30 s at 72°C. The reaction mixtures each contained 5 μl of the sample cDNA and 15 μl of the master mix, which included the appropriate sense and antisense primers. Expression of β-actin in each sample was assessed, and the β-actin data were used as an internal control to normalize differences between samples and to calculate relative expression levels. According to the minimum information for publication of quantitative real-time PCR experiments guidelines, β-actin was used as a housekeeping gene because of its high stability across porcine various tissues [20,21].

### Animals and managements

Pig were produced by crossbreeding (LWD) with Landrace (L), Large Yorkshire (W) and Duroc (D). Animals were allocated in groups of 5 heads. Piglets were taken from five different litters to perform this study. For the conformation of each experimental group, a piglet from one of each litter was selected to exclude a family effect. After weaning, all pigs were raised and fattened with the administration of a conventional diet ad libitum without supplemental antimicrobials. Pigs were grown from 3 weeks of age until week 24, and sacrificed. The group 1 (Control) was fed only the balanced conventional diet without antimicrobials ad libitum. The group 2 (Medium) was fed 200 g/day of the medium mainly contained catabolites of cow whey from 3 to 17 weeks of age. The groups 3 (*L. jensenni* TL2937) and 4 (*L. plantanum* TL2766) were fed 200 g/day of medium containing 6 × 10^10^ cfu of each Lactobacilli strains, together with conventional diet. Supplemental lactobacilli were also administered from weeks 3 to 17 of age. Body weight measurement was carried out every 2 weeks, with taking stool samples and blood. Plasma separated quickly from blood and fresh stool samples from every animal were stored at -20°C until analyzing. Carcass was also evaluated after sacrifice.

### Detection of pathogenic Escherichia coli in feces

In order to detect pathogenic *Escherichia coli* in stools, Western blotting method was carried out using anti-ETEC K88 and anti-ETEC K99 fimbrial antisera (#SSI51172, SSI51173, VERITAS Co., Tokyo), and anti-ETEC 987P fimbrial antisera (originally generated in rabbit immunized with purified pili of ETEC987P) for determination of each pili. Horseradish peroxidase conjugated anti-rabbit IgG was used as secondary antibody (#7074, Cell Signaling Technology Japan, K.K., Tokyo). All procedures followed to a commercial kit, ECL Western Blotting Detection System (GE Healthcare). Feces sample was stirred severely by sonication and separated by centrifugation for 5 min at 20°C. The precipitation was dissolved by using Thermo Scientific Tissue Protein Extraction (T-PER) Reagent (Tokyo), and purified by centrifugation. The supernatant was supplied to detection of pathogenic *Escherichia coli* in stools.

### Plasma determinations

Plasma CRP concentration was performed by using the Fujifilm clinical chemical analyzer (Fujifilm Dri-Chem 3500i, the Fujifilm Dri-Chem Slides) following the standard protocol. Plasma alternative complement activity was evaluated as disruption degree of goat red blood cell (GRBC) by pig plasma complement. A volume of 150 μL of GRBC was added gently to a mixture of 30 μL of plasma and 270 μL of experimental buffer, and then the mix was incubated at 37°C for 40 min. After the inhibition of the reaction by using 4.05 mL of EDTA solution, the supernatant was obtained by centrifugation of the mixture at 4°C for 10 min and separated quickly. Absorbance of the supernatant was determined at 542 nm of OD level.

### Blood leukocytes number

Blood leukocyte number was measured by using Celltac MEK-4100 (Nihonkohden Co.ltd.) and the specific buffers. Granulocyte/lymphocyte ratio of in peripheral blood was evaluated by determining the percentage of each leucocyte population in a smear preparation of peripheral blood sample. Smear preparations were made by using Diff-Qick stain solution. Repeated count of three times per one smear preparation was carried out by using light microscope.

### Phagocytes activity

The luminol reaction with oxygen radical occurred from broken opsonized zymosan was detected and evaluated as phagocytes activity in peripheral blood with using Fujifilm Luminescent Image Analyzer, LAS 3000. Total of luminol chemical reaction was measured sequentially and recognized as the area by the integration method. Their measurements were repeated twice per one sample. The reaction was shown as relative light unit (RLU).

### Blood antibody response

Plasma concentration of anti-GRBC IgG antibodies was measured by the ELISA method. Plates were coated with 2.5 mg/mL of rabbit anti-swine IgG diluted in phosphate-buffered saline (PBS) of pH7.2. After incubation for 2 hours at 30°C, plates were washed three times in PBS and blocked with Block Ace (DS Pharma Biomedical) for 2 hours at 30°C. Plasma samples were diluted (1:200) in PBS containing 0.05% of Tween 20, added to plates and incubated for 2 hours at 30°C. Following three washes, bound antibodies were detected with a 1:1000 dilution of affinity-purified rabbit anti-swine IgG conjugated to alkaline phosphatase conjugate, incubated for 2 hours at 30°C. After washing, the substrate p-nitrophenol phosphate was added to plates. Relative Optimal density was measured at 405 nm. The sample concentrations were calculated by reference to the linear portion of standard curve of purified swine IgG on every plate.

### Evaluation of carcass characteristics and meat quality

After sacrifice of pigs, carcass weight, oil-back fat thickness and meat quality evaluations were recorded. Carcass grading evaluation was performed based on the standards of Japanese Meat Grading Association. Carcass meats were judged by high, middle or mediocre classes and out of standards. Evaluation of tenderness, juicy and overall palatability was performed by a panel of 15 untrained persons. Pork from the different experimental groups was cooked with the same recipe and process. Panelists complete a questionnaire evaluating juicy, tenderness and overall palatability of pork. After tasting, all the dishes, the panelists were requested to grade taste based on three categories: distasteful, acceptable and extremely delicious.

### Statistical analysis

Statistical analysis was performed by using SAS programs (Version 9.1). Relative indices were calculated respectively as the ratio of cytokine mRNA expression to beta-actin. Relative indices were respectively normalized by common logarithmic transformation and confirmed as approximate value included significantly into normal distribution. They were adjusted similarly that means of the control group were adjusted to 1.0 with standard deviations (SD). In all items, all of means and SDs were calculated by each 3 repeated measurements by category. To examine the significance for fixed effect among experiment's conditions, one-way ANOVA was carried out. To examine the significance for a fixed effect among experiment's conditions, one-way ANOVA was carried out (Additional file 1: Table S1). And then Duncan's method for multi-comparison was performed to compare among means of every category at 5% significance level.

## Results

### Lactobacillus jensenii TL2937 modulates cytokines production in porcine intestinal epithelial cells - antigen presenting cells co-cultures

**Figure 1:**
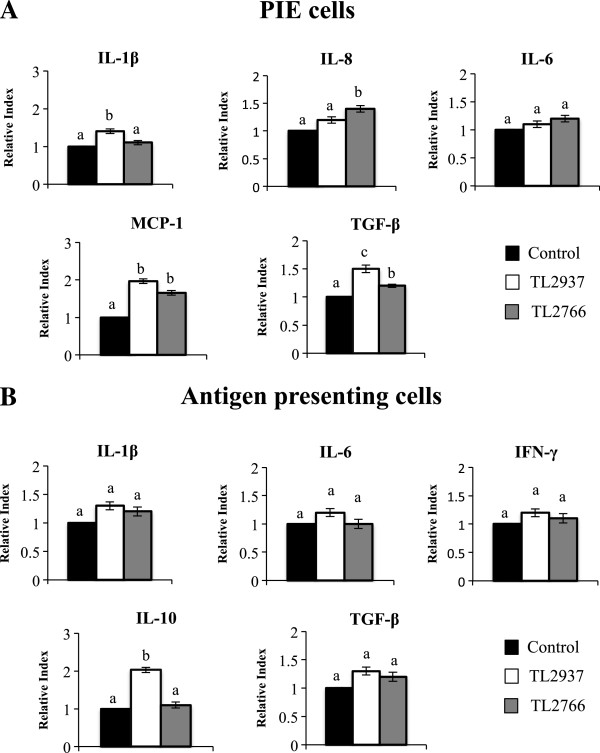
**Effect of *Lactobacillus jensenii *TL2937 on porcine intestinal epithelial (PIE) cells co-cultured with adherent population from swine Peyer’s Patches (PPs).** Antigen-presenting cells (macrophages and dendritic cells) from PPs were obtained after their adherence to glass. PIE-Adherent cells co-cultures were treated with *L. jensenii* TL2937 or *L. plantarum* TL2966 for 48 h. Untreated PIE-adherent cells co-cultures were used as controls. **(A)** Expression of IL-1β, IL-8, IL-6, MCP-I, and TGF-β mRNAs was examined in PIE cells using RT-qPCR. **(B)** Expression of IL-1β, IL-6, IL-10, IFN-γ, and TGF-β mRNAs was examined in adherent cells using RT-qPCR. Values for bars with different letters were significantly different (P < 0.05). Values for bars with shared letters do not differ significantly. The results represent data from three independent experiments using ileal PPs from at least three different swine.

We evaluated the effect of *L. jensenii* TL2937 (anti-inflammatory strain) and *L. plantarum* TL2766 (negative control) in PIE-APCs co-cultures. We previously demonstrated that TLR2 is important for the immunoregulatry effects of the TL2937 strain in PIE [9] and APCs [10]. Then, *L. plantarum* TL2766 was selected as a negative control considering its incapacity to efficiently activate TLR2 [9,10]. PIE-APCs co-cultures were separately stimulated with each of two lactobacillus strains for 48 hours and the levels of IL-1β, IL-6, IL-8, MCP-1 and TGF-β were determined in PIE cells. As previously described [9] lactobacilli did not modify PIE cell viability, because more than 95% of PIE cells were viable in all cases (data not shown). *L. jensenii* TL2937 increased mRNA IL-1β expression while the TL2766 strain did not modified this cytokine in PIE cells (Figure 1A). On the contrary, IL-8 mRNA levels were upregulated by *L. plantarum* TL2766 while the TL2937 strain did not change the expression of this chemokine. IL-6 was not significantly modified by the lactobacilli, in contrast both *L. jensenii* TL2937 and *L. plantarum* TL2766 upregulated MCP-1 and TGF-β mRNA levels in PIE cells (Figure 1A).

**Figure 2:**
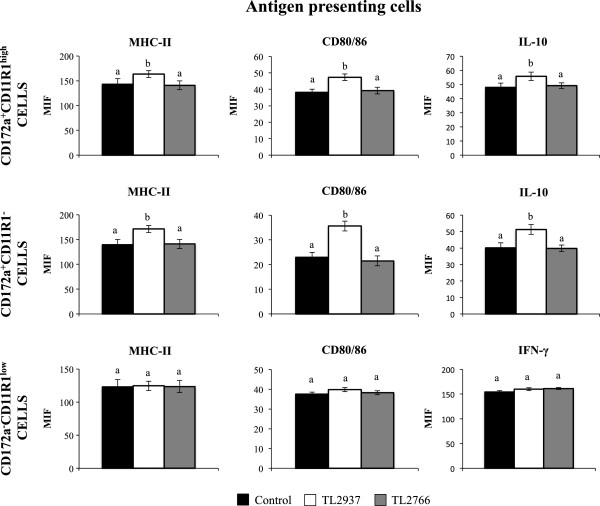
**Effect of *Lactobacillus jensenii *TL2937 on porcine intestinal epithelial (PIE) cells co-cultured with adherent population from swine Peyer’s Patches (PPs).** Antigen-presenting cells (macrophages and dendritic cells) from PPs were obtained by taking advantage of their capacity to adhere to glass. PIE-Adherent cells co-cultures were treated with *L. jensenii* TL2937 or *L. plantarum* TL2966 for 48 h. Untreated PIE-adherent cells co-cultures were used as controls. Expression of MHC-II, CD80/86, IL-10, and IFN-γ was studied in CD172a^+^CD11R1^-^, CD172a^+^CD11R1^high^, and CD172a^-^CD11R1^low^ adherent cells by flow cytometric analysis. Values for bars with different letters were significantly different (P < 0.05). Values for bars with shared letters do not differ significantly. The results represent data from three independent experiments using ileal PPs from at least three different swine.

In addition, we evaluated the expression of several cytokines in APC co-cultured with lactobacilli stimulated-PIE cells, evaluating in this way the indirect effect of lactobacilli on immune cells. The stimulation of PIE-APCs co-cultures with *L. jensenii* TL2937 did not induce significant changes in expression of TNF-α, IL-2, IL-4, IL-12 (data not shown) or IL-6, IL-1β, TGF-β and IFN-γ in APCs (Figure 1B). However, in APCs co-cultured with *L. jensenii* TL2937-treated PIE cells the expression of IL-10 was 2.03-fold higher than controls (Figure 1B). No changes were observed in cytokines expression in APCs from PIE-APCs co-cultures treated with *L. plantarum* TL2766 (Figure 1B). As we described previously [10], we are able to define three populations of APCs in adherent cells from PPs by using anti-CD172a and anti-CD11R1 antibodies: CD172a^+^CD11R1^-^, CD172a^+^CD11R1^high^, and CD172a^-^CD11R1^low^ cells which exhibit strong expression of MHC-II. In addition, we demonstrated previously that *L. jensenii* TL2937 is able to upregulate the expression of IL-10 in CD172a^+^ cells and the expression of IFN-γ CD172a^-^ cells [10]. In this work, we observed that stimulation of PIE-APCs co-cultures with *L. jensenii* TL2937 increased the expression of MHC-II, CD80/86 and IL-10 in CD172a^+^CD11R1^-^ and CD172a^+^CD11R1^high^ cells, while no changes were observed in the expression of MHC-II, CD80/86 and IFN-γ in CD172a^-^CD11R1^low^ cells (Figure 2). No modifications in the levels of MHC-II, CD80/86, IL-10 or IFN-γ were observed in APCs from PIE-APCs co-cultures treated with *L. plantarum* TL2766 (Figure 2).

### Lactobacillus jensenii TL2937 differentially modulates the inflammatory response against ETEC in porcine intestinal epithelial cells - antigen presenting cells co-cultures

**Figure 3:**
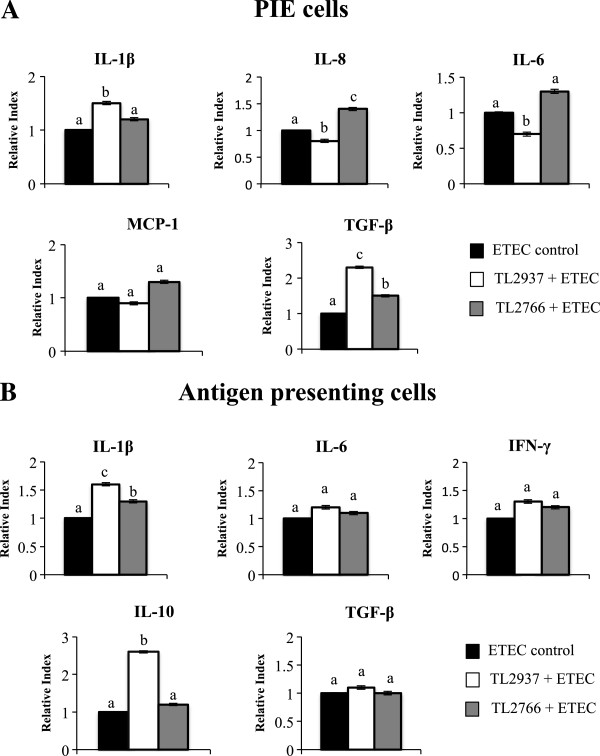
**Effect of *Lactobacillus jensenii *TL2937 on porcine intestinal epithelial (PIE) cells co-cultured with adherent population from swine Peyer’s Patches (PPs) under inflammatory conditions.** PIE-Adherent cells co-cultures were treated with *L. jensenii* TL2937 or *L. plantarum* TL2966 for 48 h. Untreated PIE-adherent cells co-cultures were used as controls. After lactobacilli stimulation, treated cells and untreated controls were challenge with ETEC (5 × 10^7^ cells/ml). **(A)** Expression of IL-1β, IL-8, IL-6, MCP-I, and TGF-β mRNAs was examined in PIE cells using RT-qPCR. **(B)** Expression of IL-1β, IL-6, IL-10, IFN-γ, and TGF-β mRNAs was examined in adherent cells using RT-qPCR. Values for bars with different letters were significantly different (P < 0.05). Values for bars with shared letters do not differ significantly. The results represent data from three independent experiments using ileal PPs from at least three different swine.

The anti-inflammatory capacities of *L. jensenii* TL2937 and *L. plantarum* TL2766 were evaluated in PIE-APCs co-cultures challenged with ETEC (Figure 3). Individual PIE-APCs co-cultures were stimulated with a single lactobacilli strain for 48 hours and then challenged with ETEC. The levels of IL-1β, IL-6, IL-8, and MCP-1 mRNAs in PIE cells were studied 12 hours after the challenge. Stimulation with the intestinal pathogen resulted in significant increases of pro-inflammatory cytokine expression in lactobacillus-treated and untreated control PIE cells (Figure 3A). However, IL-6 and IL-8 mRNA levels in PIE cells stimulated with the TL2937 strain were significantly lower than those observed in the ETEC control. In contrast, IL-1β and TGF-β were significantly higher in TL2937-treated PIE cells than the controls while MCP-1 mRNA levels in PIE cells treated with *L. jensenii* TL2937 were not different from the ETEC control (Figure 3A). *L. plantarum* TL2766 did not induced significant changes in the expression of cytokines after the challenge with ETEC with the exception of TGF-β and IL-8 levels that were significantly higher in TL2766-treated PIE cells than in controls (Figure 3A).

**Figure 4:**
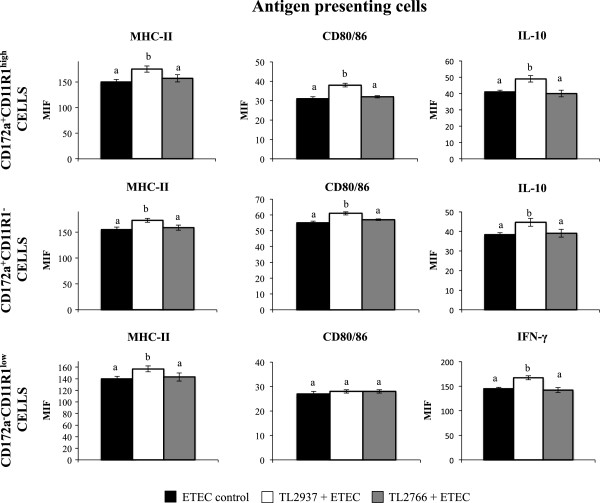
**Effect of *Lactobacillus jensenii *TL2937 on porcine intestinal epithelial (PIE) cells co-cultured with adherent population from swine Peyer’s Patches (PPs) under inflammatory conditions.** PIE-Adherent cells co-cultures were treated with *L. jensenii* TL2937 or *L. plantarum* TL2966 for 48 h. Untreated PIE-adherent cells co-cultures were used as controls. After lactobacilli stimulation, treated cells and untreated controls were challenge with ETEC (5 × 10^7^ cells/ml). Expression of MHC-II, CD80/86, IL-10, and IFN-γ was studied in CD172a^+^CD11R1^-^, CD172a^+^CD11R1^high^, and CD172a^-^CD11R1^low^ adherent cells by flow cytometric analysis. Values for bars with different letters were significantly different (P < 0.05). Values for bars with shared letters do not differ significantly. The results represent data from three independent experiments using ileal PPs from at least three different swine.

In addition, the mRNA levels of various cytokines were assessed in APCs 12 hours after the challenge of PIE-APCs co-cultures with ETEC (Figure 3B). The challenge with ETEC resulted in an increase in the expression of TNF-α, IL-2, IL-12 (data not shown), IL-1β, IL-6 and IFN-γ (Figure 3B); in contrast, the ETEC stimulation induced a decrease in the expression of the immunomodulatory cytokines TGF-β and IL-10 (Figure 3B). Pretreatment of PIE-APCs co-cultures with *L. jensenii* TL2937 did not induce significant changes in the levels of TNF-α, IL-2, IL-12 (data not shown), TGF-β, IL-6 and IFN-γ in APCs after the challenge with ETEC (Figure 3B). However, the TL2937 strain did induce higher levels of IL-10 and IL-1β in APCs co-cultured with PIE cells (Figure 3B). APCs in PIE-APCs co-cultures previously stimulated with *L. plantarum* TL2766 showed increased levels of IL-1β after the stimulation with ETEC while the other cytokines studied were not modified when compared to controls (Figure 3B). Challenge of PIE-APCs co-cultures with ETEC resulted in significant increases in the expression of MHC-II and CD80/86 in all populations of APCs. However, in CD172a^+^CD11R1^-^, CD172a^-^CD11R1^low^ and CD172a^+^CD11R1^high^ cells pretreated with *L. jensenii* TL2937, the levels of MHC-II were higher than those observed in ETEC control cells (Figure 4). In addition, levels of CD80/86 in CD172a^+^CD11R1^-^ and CD172a^+^CD11R1^high^ cells from PIE-APCs co-cultures treated with the TL2937 strain were higher than those observed in ETEC control cells (Figure 4). IL-10 levels were significantly higher in CD172a^+^CD11R1^-^ and CD172a^+^CD11R1^high^ cells and pretreated with *L. jensenii* TL2937 (Figure 4). IFN-γ in CD172a^-^CD11R1^low^ was slightly but significant higher than control cells (Figure 4). No modifications in the levels of MHC-II, CD80/86, IL-10 or IFN-γ were observed in APCs from PIE-APCs co-cultures treated with *L. plantarum* TL2766 when compared to untreated controls (Figure 4).

### Lactobacillus jensenii TL2937 modulates the expression of negative regulators of Toll-like receptors in porcine intestinal epithelial cells - antigen presenting cells co-cultures

**Figure 5:**
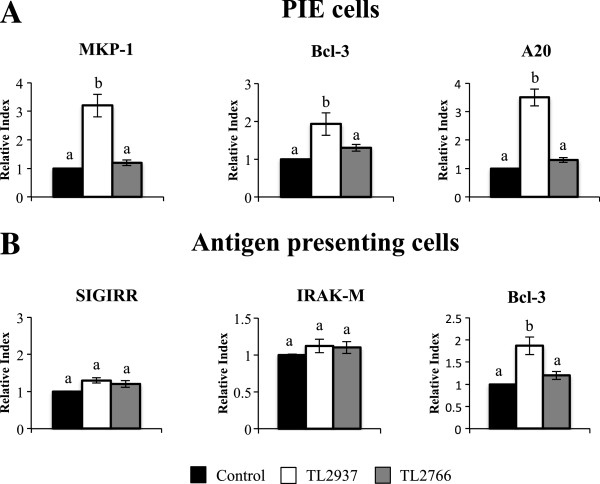
**Effect of *Lactobacillus jensenii *TL2937 on negative regulators of the TLR signaling pathway in porcine intestinal epithelial (PIE) cells co-cultured with adherent population from swine Peyer’s Patches (PPs).** PIE-Adherent cells co-cultures were treated with *L. jensenii* TL2937 or *L. plantarum* TL2966 for 48 h. Untreated PIE-adherent cells co-cultures were used as controls. Expression of SIGIRR, A20, Bcl-3, MKP-1, and IRAK-M mRNAs was measured in **(A)** PIE cells and **(B)** adherent cells using RT-qPCR. Values for bars with different letters were significantly different (P < 0.05). Values for bars with shared letters do not differ significantly. The results represent data from three independent experiments using ileal PPs from at least three different swine.

We next studied the effect of lactobacilli on the expression of six regulators that inhibit the TLR signaling pathway: SIGIRR, Tollip, A20, Bcl-3, MKP-1, and IRAK-M in both PIE cells and APCs (Figure 5). PIE-APCs co-cultures were stimulated for 48 hours with *L. jensenii* TL2937 or *L. plantarum* TL2766 and the expression levels of SIGIRR, Tollip, A20, Bcl-3, MKP-1, and IRAK-M mRNA were determined using real-time PCR as previously described [9,10]. None of the lactobacillus strains induced changes in expression of SIGIRR, Tollip, or IRAK-M in PIE cells (data not shown). However, *L. jensenii* TL2937 caused upregulation of MKP-1, Bcl-3 and A20 mRNA levels in PIE cells (Figure 5A). No significant changes in SIGIRR, Tollip, A20, Bcl-3, MKP-1 or IRAK-M mRNA expression were observed in PIE cell treated with *L. plantarum* TL2766 (Figure 5A). In addition, treatment of PIE-APCs co-cultures with *L. jensenii* TL2937 resulted in upregulation of the expression of Bcl-3 in APCs (Figure 5B) while no modifications in the expression of the other TLR negative regulators were observed. There were also no changes in expression of any of these negative regulators of TLRs in the studies in which we employed *L. plantarum* TL2766 (Figure 5B).

### Lactobacillus jensenii TL2937 improves immune-health status of piglets

**Figure 6:**
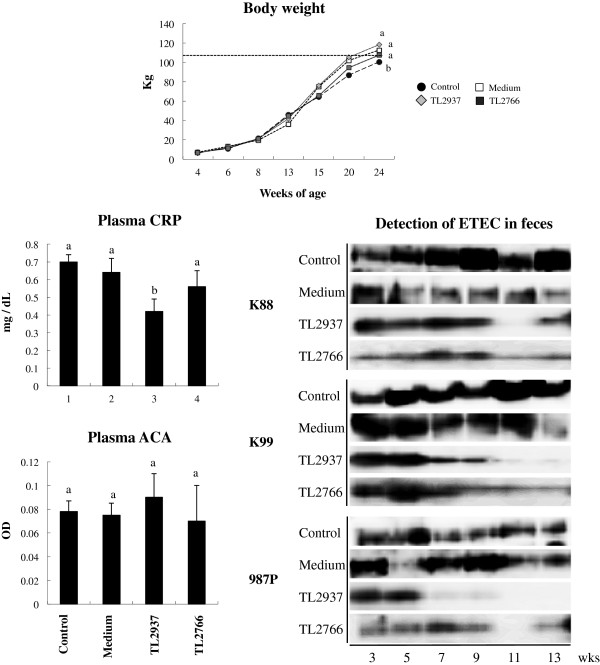
**Effect of *Lactobacillus jensenii *TL2937 on piglets’ growth and immune health.** Pigs were grown from 3 weeks of age until week 24. Five pigs were used for each experimental group. The Control group was fed only the balanced conventional diet without antimicrobials ad libitum. The Medium, TL2937 and TL2766 groups were fed balanced conventional diet with supplemental bacteria medium only (200 g/day), *L. jensenni* TL2937 (3 × 10^8^ cfu/g) or *L. plantanum* TL2766 (3 × 10^8^ cfu/g) respectively, from 3 to 17 weeks of age. Body weight measurement, detection of enterotoxigenic *Escherichia coli* in feces, and plasma levels of alternative complement activity (ACA) and C reactive protein (CRP) were determined every 2 weeks. The line in body weight indicates the suitable body weight for shipping in Japan. Values for bars with different letters were significantly different (P < 0.05). Values for bars with shared letters do not differ significantly.

Our previous reports [9,10] and the studies presented above clearly indicated that *L. jensenii* TL2937 has a high potential to beneficially modulate mucosal immune system in piglets and improve health. Therefore, we next aimed to demonstrate in vivo the immunobiotic effect of the TL2937 strain. For this purpose five piglets of 3 weeks of age were fed *L. jensenii* TL2937 until they reached the age of 15 weeks. *L. plantarum* TL2766 was used as a negative control and was administered to a second group of five piglets during the same period. In addition, untreated and medium-treated piglets were used as controls. Body weight changes were recorded until week 24 of age as shown in Figure 6. As expected, control pigs reached 115 kg of weight in week 24. However, piglets fed the TL2937 strain reached this suitable body weight earlier than controls. This difference of almost 4 weeks represents in did an improvement of growth performance and productivity of piglets. Surprisingly, administration of *L. plantarum* TL2766 also significantly increased the body weight of pigs on week 24 when compared to controls (Figure 6). In addition, no differences were found between the groups when comparing plasma levels of Free Fatty Acid (FFA), glucose, triglycerides or cholesterol (Additional file 2: Figure S1). Analysis of the presence of ETEC in pigs’ feces at week 13 demonstrated an apparent reduction in the recovery of K88, K99 and 987P ETEC strains in TL2937-treated pigs while *L. plantarum* TL2766 treatment reduced the presence of 987P ETEC only (Figure 6). In order to evaluate the general inflammatory status of pigs, alternative complement activity (ACA) and C reactive protein (CRP) levels were determined in plasma (Figure 6). No significant changes were observed in plasma ACA after the lactobacilli treatments; however, treatment with *L. jensenii* TL2937 significantly reduced plasma CRP to normal level (Figure 6). No changes were observed in blood leukocytes, ratio of granulocytes to lymphocyte numbers, phagocytes activity or antibody levels in *L. jensenii* TL2937 and *L. plantarum* TL2766 treated pigs when compared to controls (Additional file 3: Figure S2).

### Lactobacillus jensenii TL2937 increases productivity of piglets

**Figure 7:**
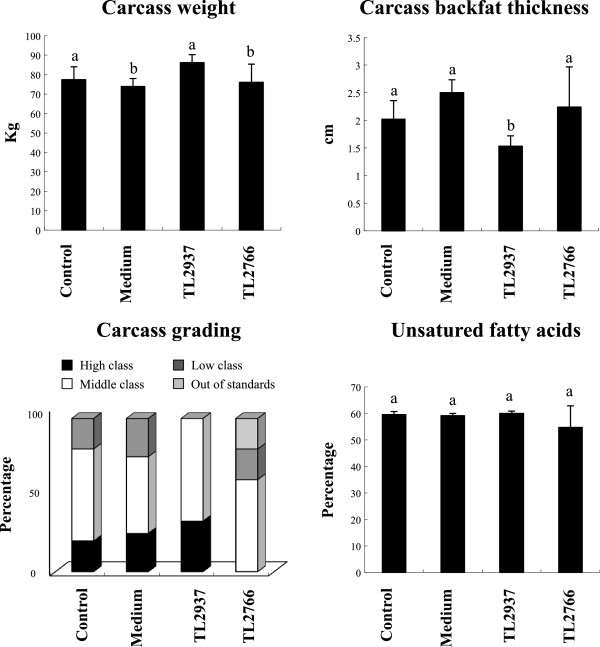
**Effect of *Lactobacillus jensenii *TL2937 on carcass quality.** Pigs were grown from 3 weeks of age until week 24. Five pigs were used for each experimental group. The Control group was fed only the balanced conventional diet without antimicrobials ad libitum. The Medium, TL2937 and TL2766 groups were fed balanced conventional diet with supplemental bacteria medium only (200 g/day), *L. jensenni* TL2937 (3 × 10^8^ cfu/g) or *L. plantanum* TL2766 (3 × 10^8^ cfu/g) respectively, from 3 to 17 weeks of age. After sacrifice of pigs, carcass weight, oil-back fat thickness and unsaturated fatty acids were evaluated. Carcass grading evaluation was performed based on the standards of Japanese Meat Grading Association. Carcass meats were judged by high, middle or mediocre classes and out of standards. Values for bars with different letters were significantly different (P < 0.05). Values for bars with shared letters do not differ significantly.

**Figure 8:**
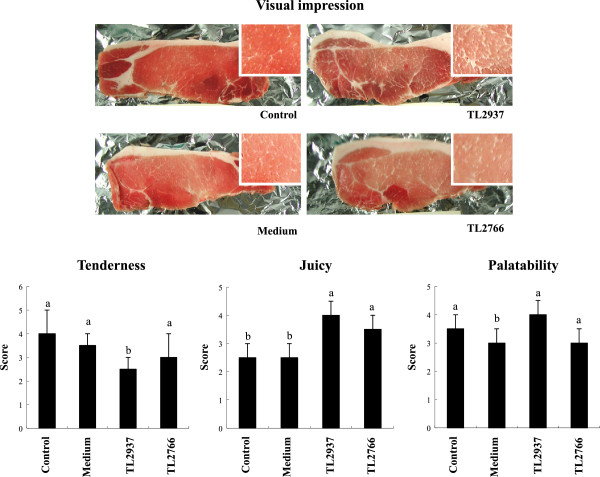
**Effect of *Lactobacillus jensenii *TL2937 on meat quality.** Pigs were grown from 3 weeks of age until week 24. Five pigs were used for each experimental group. The Control group was fed only the balanced conventional diet without antimicrobials ad libitum. The Medium, TL2937 and TL2766 groups were fed balanced conventional diet with supplemental bacteria medium only (200 g/day), *L. jensenni* TL2937 (3 × 10^8^ cfu/g) or *L. plantanum* TL2766 (3 × 10^8^ cfu/g) respectively, from 3 to 17 weeks of age. Visual impression of pork meat and evaluation of tenderness, juicy and overall palatability was performed by a panel of untrained persons. Pork from the different experimental groups was cooked with the same recipe and process. Panelists complete a questionnaire evaluating juicy, tenderness and overall palatability of pork. After tasting, all the dishes, the panelists were requested to grade taste based on three categories: distasteful, acceptable and extremely delicious. Values for bars with different letters were significantly different (P < 0.05). Values for bars with shared letters do not differ significantly.

We also evaluated carcass weight and backfat thickness as shown in Figure 7. No significant differences were observed in carcass weight after the treatment with lactobacilli; however, carcass backfat thickness was significantly reduced in pigs receiving *L. jensenii* TL2937 when compared to controls and those treated with the TL2766 strain (Figure 7). In addition, carcass grading evaluation was performed according to the standards of the Japanese Meat Grading Association that considers four grades of carcass grading as shown in Figure 7. In control pigs, 20, 60 and 20% of carcass corresponded to high, middle and low class quality respectively (Figure 7). Administration of medium did not induce changes with respect to controls; however, *L. jensenii* TL2937 treatment significantly increased carcass grading. In TL2937-treated pigs 33 and 77% of carcass corresponded to high and middle class quality respectively (Figure 7). Surprisingly, treatment with *L. plantarum* TL2766 negatively affected carcass grading. In this group, 60 and 20% of carcass corresponded to middle and low class quality respectively, while 20% was out of standards (Figure 7).Considering that fresh meat color and appearance has a subtle but important impact on consumers’ decisions, next a panel of 15 persons blindly performed a visual evaluation of pork meat from each of the four experimental groups, considering marbling (intramuscular fat), color, firmness and wetness (Figure 8). Pork meat from the control group showed low intramuscular fat and a bright red color. Moreover, control meat was firm and dry. Medium group was not different from control group (Figure 8). On the other hand, in the TL2937 group meat was light red and bright, whit high intramuscular fat, soft and juicy (Figure 8); while in the TL2677 group meat was opaque and with a light red color. Meat from TL2677 pigs has low intramuscular fat (Figure 8).

In addition, pork meat coming from the different experimental groups was cooked with the same process and juicy, tenderness, and overall palatability was evaluated by panelists. *L. jensenii* TL2937 administration significantly reduced tenderness and improved juicy and palatability of pork meat when compared to controls and medium-treated pigs (Figure 8). Tenderness, juicy and palatability in TL2677-treated pigs were not different from controls (Figure 8).

## Discussion

Several works have demonstrated that microbial recognition by IECs is an integral aspect of first-line host responses, pointing to the idea that the epithelium is more than simply a physical barrier that separates luminal contents from mucosal APCs [11-13]. The intestinal epithelium is increasingly recognized as playing an essential role in immune homeostasis, through the promotion of tolerogenic and regulatory responses. These findings have important implications for the regulation of mucosal homeostasis by probiotic bacteria. The utilization of in vitro systems allowing conditioning of immune cells through co-culture with IECs has demonstrated that probiotic-induced signaling in IECs plays an essential role in the immunoregulatory effect of some immunobiotic strains [9,18,19,22,23].

In the present work we evaluated the immunoregulatory effect of *L. jensenii* TL2937 by using an in vitro porcine PIE-APCs co-culture system. We observed a significant upregulation of proinflammatory mediators in PIE cells co-cultured with adherent cells and challenged with ETEC. This finding was consistent with findings from a previous study demonstrating that PIE monocultures induce inflammatory responses by upregulating cytokines and chemokines in response to ETEC challenge [9]. Therefore, we demonstrated that PIE cells did not respond differently to ETEC challenge when co-cultured with APCS. Moreover, our present results confirmed that the pretreatment of PIE cells with *L. jensenii* TL2937 was able to reduce levels of proinflammatory cytokines in response to ETEC and that this effect was related to upregulation of three TLR negative regulators: A20, Bcl-3, and MKP-1 as in PIE cell monocultures [9]. In addition, we demonstrated for the first time in this work that *L. jensenii* TL2937 is able to induce the expression TGF-β in PIE cells. It is well known that IECs-derived factors are able to condition mucosal DCs, especially the cells of the CD11b^+^ subset, to secrete cytokines such as IL-10 and TGF-β in response to commensal microbes, thereby initiating differentiation of Treg immune responses [24]. Moreover, conditioning of monocyte-derived DCs with IECs supernatants confer on DCs the capacity to produce large amounts of IL-10, which is attributable, at least in part, to the release of the IECs-derived factors such as TGF-β and thymic stromal-derived lymphopoietin (TSLP) [25]. Therefore, in addition to its direct tolerogenic effects on PIE cells [9] and APCs [10], *L. jensenii* TL2937 could have an indirect anti-inflammatory effect on APCs under the influence of factors produced by PIE cells such as TGF-β.

When we studied the indirect effect of *L. jensenii* TL2937 on APCs in co-cultures with PIE cells, we observed that the response of APCs was completely different to those observed in APCs monocultures [10]. Previously, we demonstrated that direct exposure of porcine APCs to *L. jensenii* TL2937 increased the expression of IL-10 and TGF-β in CD172a^+^CD11R1^-^ and CD172a^+^CD11R1^high^ cells, while the treatment with this bacterium was associated with increased levels of IFN-γ in CD172a^-^CD11R1^low^ adherent cells from PPs [10]. We also evaluated in a previous work the effect of the TL2937 strain on the expression of negative regulators of TLRs in APCs. Of the six regulators tested, SIGIRR, A20, and IRAK-M mRNA expression was upregulated in APCs cells stimulated with *L. jensenii* TL2937. These changes resulted in differential modulation of the production of pro- and anti-inflammatory cytokines in response to ETEC or LPS challenges [10]. In PIE-APCs co-cultures, no modifications in the levels of TGF-β in CD172a^+^CD11R1^-^ and CD172a^+^CD11R1^high^ cells or levels of IFN-γ in CD172a^-^CD11R1^low^ cells were observed. However, increased levels of IL-10 were observed in CD172a^+^ cells co-cultured with PIE cells. In addition, no modification in SIGIRR, A20 or IRAK-M expression was observed in this work. Notably, Bcl-3 expression was upregulated in APCs cells co-cultured with PIE cells. The Bcl-3 protein functions as an inhibitor of NF-κB activity. It was reported that treatment of macrophages with IL-10 induces the expression of Bcl-3, and Bcl-3 expression leads to inhibition of LPS-induced TNF-α production [26]. Then it is probable that immunoregulatory cytokines (IL-10) produced by APCs act in an autocrine way and upregulate the expression Bcl-3. Then, the results presented here demonstrate that the response of PPs APCs to *L. jensenii* TL2937 is significantly modified when the stimulus is mediated indirectly through IECs.

**Figure 9:**
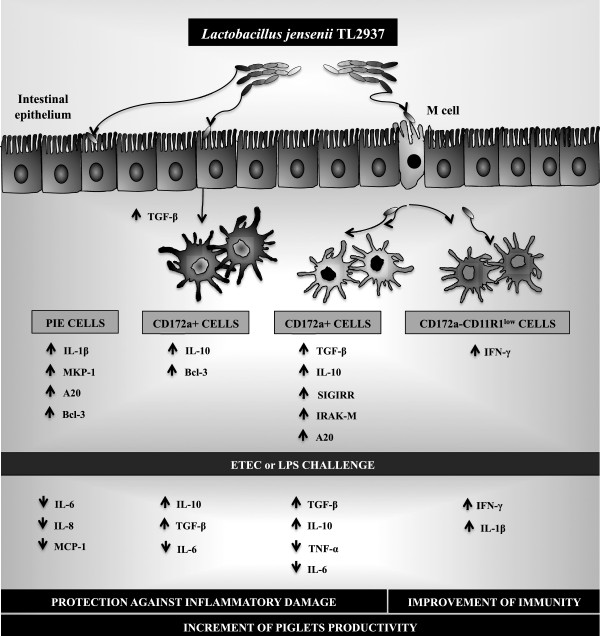
**Proposed mechanism for the immunomodulatory effect of *Lactobacillus jensenii *TL2937 on porcine intestinal mucosa.** B-cell lymphoma 3-encoded protein (Bcl-3), Enterotoxigenic *Escherichia coli* (ETEC), Interferon (IFN)-γ, Interleukin (IL), Interleukin-1 receptor-associated kinase M (IRAK-M), Mitogen- activated protein kinase (MAPK), Mitogen-activated protein kinase 1 (MPK-1), Monocyte chemotactic protein-1 (MCP-1), Nuclear factor κB (NF-κB), Single immunoglobulin IL-1-related receptor (SIGIRR), Toll-like receptor (TLR)-4, Transforming growth factor (TGF)-β, Tumor necrosis factor (TNF), Ubiquitin-editing enzyme A20 (A20).

Our previous results and the ones presented here, allow us to propose a more complete view of the cellular and molecular mechanisms involved in the immunoregulatory effects of *L. jensenii* TL2937 (Figure 9). When reaching the porcine intestinal mucosa, *L. jensenii* TL2937 would have the capacity to interact with local cells at three levels: first, the interaction of the TL2937 strain with IECs would induce the upregulation of MKP-1, Bcl3 and A20 expression [9,22]. Second, *L. jensenii* TL2937 could be taken by APCs indirectly through M cell transport or by direct sampling from the intestinal lumen, inducing an increase in the production of the immunoregulatory cytokines IL-10 and TGF-β by CD172a^+^CD11R1^-^ and CD172a^+^CD11R1^high^ cells as well as the expression of SIGIRR, IRAK-M and A20. In addition, through its direct interaction with CD172a^-^CD11R1^low^ cells, the TL2937 strain would have the capacity to improve Th1 responses by increasing the production of IFN-γ [10]. Finally, as demonstrated here, *L. jensenii* TL2937 would be able of stimulating the production of immunoregulatory factors such as TGF-β in EICs, which would increase the expression of Bcl-3 and the production of IL-10 in CD172a^+^ APCs. Then, *L. jensenii* TL2937 would functionally modulate IECs and APCs to improve resistance against infections and avoid non-protective inflammation. In fact, our in vitro experiments using ETEC challenge, clearly demonstrated that the TL2937 strain is able to induce protection against inflammatory damage and improve immunity at the same time (Figure 9) [9,10].

In addition, in this study we provide original in vivo data concerning the immunoregulatory effect of *L. jensenii* TL2937. We demonstrated that the administration of *L. jensenii* TL2937 significantly increased grow performance and productivity of piglets, an effect that could be related to the improvement of immune-health. As mentioned before, at weaning, piglets are stressed, the food intake is strongly depressed, the structure and function of the gastrointestinal tract are altered, and these conditions can favor bacterial translocation, inflammation and infection with pathogenic bacteria [27,28]. This adaptation of piglets to new environments during early life has profound effects on intestinal microbiota, especially on its metabolic activities and immunoregulatory functions. These changes in intestinal microbiota require special consideration when viewed in the context of pig production in which efficiency of animal growth is a primary objective. It was demonstrated that the optimal gut microbiota significantly improves intestinal health and beneficially affects the efficiency of gastrointestinal and whole body growth throughout the productive life cycle of a pig [28]. In this regard, studies of the transcriptome profiling of the small intestinal epithelium in germfree versus conventional neonatal piglets showed higher levels of TOLLIP and NF-κBIA, a protein associated with the inactivation of NF-κB by sequestration, in colonized versus germ free animals [29]. Moreover, gut microbiota of the ileal epithelium was associated with a downregulation of GATA1 [29]. These findings reflect the role of gut microbiota in the activation of pathways that prevent excessive inflammation [29]. In addition, it is extremely important to direct piglets intestinal immune system toward appropriate immune responses that strives to maintain intestinal homeostasis, not only in the induction of tolerance against harmful antigens, but in effective effectors responses against pathogens. Some studies have associated probiotic bacteria with the improvement of intestinal homeostasis in pigs, albeit with different levels of success [30,31].

Recent studies by Li et al. [30] showed that pretreatment of piglets with *L. rhamnosus* ATCC7469 ameliorates F4^+^ETEC-induced diarrhoea. In that study, jejunal TLR4 expression at the mRNA and protein levels, and jejunal IL-8 mRNA expression were significantly elevated in piglets exposed to F4^+^ETEC; and the increased intestinal TLR4 and IL-8 mRNA expression was attenuated by pretreatment with the ATCC7469 strain. Moreover, authors showed an induction of ileal NOD1 that was accompanied by upregulation of TLR2 and TLR9 expression in the pigs pretreated with *L. rhamnosus*, suggesting that the anti-inflammatory effect of the ATCC7469 strain may be a result of synergistic responses of TLR2, TLR9 and NOD1. Our in vivo experiments indicate that *L. jensenii* TL2937 would exerts a similar effect in pigs, since we found reduced markers of inflammation in blood. Then, the immunobiotic TL2937 strain would be able to avoid non-protective inflammation. Moreover, our in vitro experiments also indicate that these effects are mainly related to TLR2 activation although other PPRs could be involved [9,10] similarly to the work of Li et al. [30].

The capacity to modulate the immune system seems to be related to the complex secretion of cytokines induced by probiotics in the gut. *L. jensenii* TL2937 could strongly induced secretion of IL-10 and IFN-γ which would be related to the beneficial effects achieved by the immunobiotic strain, although more cytokines could be also involved. In line with these findings, it was demonstrated that administration of *Bifidobacterium longum* AH1206 to suckling piglets caused a dose-dependent linear increase in both mucosal IL-10 and TNF-α mRNA expression, indicating the potential for modulation of the inflammatory tone of the intestinal mucosa and the improvement of defenses against pathogens [32]. The capacity to modulate inflammation and improve defences at the same time has been described for several probiotic strains [33-35]. *L. jensenii* TL2937 could be included in the list of probiotic strains with those capabilities, however further experiments using controlled pathogen-challenge experiments are necessary to demonstrate the anti-pathogenic activities of this strain.

## Conclusion

Post-weaning diarrhoea mainly occurs within the first week after weaning and affects pigs across the globe, causing great economic loss to the swine industry due to reduced growth performance and considerable morbidity and mortality. We demonstrated here that the use of immunobiotics strains as supplemental additives for piglets feedings could be used as a strategy to maintain and improve intestinal homeostasis; that is important for the development of the pig and for health and performance throughout the productive life of the animal.

The scientific research into probiotic mode of actions has come to age and has shown how probiotics are able to induce beneficial changes in the host. In this study, we provide new in vitro and in vivo data to propose a more complete a view of the cellular and molecular mechanisms involved in the immunoregulatory effects of *L. jensenii* TL2937. The previous and the present results indicate that the immunological networks induced by *L. jensenii* TL2937 would help to maintain intestinal tolerance and improve the development of appropriate protective and controlled immune responses. Then, *L. jensenii* TL2937 has a great potential to be used as a pig probiotic feed.

## Competing interests

We have the following interests: Seiya Makino and Shuji Ikegami are employed by Meiji Co., Ltd. There are no patents, products in development or marketed products to declare. This does not alter our adherence to all the BMC Immunology policies on sharing data and materials.

## Authors' contributions

YS, JV, YT, SH, YT, KT, TS, MT, MI, SM, and SI carried out experiments, analyzed data and performed the statistical analysis. YS, JV, HA and HK conceived of the study, and participated in its design and coordination and helped to draft the manuscript. All authors read and approved the final manuscript.

## Supplementary Material

Additional file 1: Table S1 Analysis of variance. Click here for file

Additional file 2: Figure S1 Effect of *Lactobacillus jensenii* TL2937 on piglets’ plasma biochemical markers. Pigs were grown from 3 weeks of age until week 24. Five pigs were used for each experimental group. The Control group was fed only the balanced conventional diet without antimicrobials ad libitum. The Medium, TL2937 and TL2766 groups were fed balanced conventional diet with supplemental bacteria medium only (200 g/day), *L. jensenni* TL2937 (3 × 10^8^ cfu/g) or *L. plantanum* TL2766 (3 × 10^8^ cfu/g) respectively, from 3 to 17 weeks of age. Plasma levels of Free Fatty Acid (FFA), glucose, total cholesterol (TC) and triglycerides (TG) were determined at the end of experiments. Values for bars with different letters were significantly different (P < 0.05). Values for bars with shared letters do not differ significantly. Click here for file

Additional file 3: Figure S2 Effect of *Lactobacillus jensenii* TL2937 on piglets’ blood immune system markers. Pigs were grown from 3 weeks of age until week 24. Five pigs were used for each experimental group. The Control group was fed only the balanced conventional diet without antimicrobials ad libitum. The Medium, TL2937 and TL2766 groups were fed balanced conventional diet with supplemental bacteria medium only (200 g/day), *L. jensenni* TL2937 (3 × 10^8^ cfu/g) or *L. plantanum* TL2766 (3 × 10^8^ cfu/g) respectively, from 3 to 17 weeks of age. Levels of blood leucocytes, granulocytes/lymphocytes ratio, phagocytes activity, and antibodies were determined at the end of experiments. Values for bars with different letters were significantly different (P < 0.05). Values for bars with shared letters do not differ significantly. Click here for file
