# Supplementary material for: Immunobiotic Lactobacillus jensenii as immune-health promoting factor to improve growth performance and productivity in post-weaning pigs
Source: BMC Immunol. 2014 Jun 19;15:24. doi: 10.1186/1471-2172-15-24 (PMC4068960; doi:10.1186/1471-2172-15-24)
Supplement: Additional file 1: Table S1 — Analysis of variance. [file 1471-2172-15-24-S1.pdf]

Supplementary Table 1. Analysis of variance.

Item	Fig	Effects for experiment's conditions	P value
IL-1 β	1A	Control, TL2937, TL2766	<0.05
IL-8	1A	Control, TL2937, TL2766	<0.05
IL-6	1A	Control, TL2937, TL2766	N.S.
MCP-1	1A	Control, TL2937, TL2766	<0.05
TGF- β	1A	Control, TL2937, TL2766	<0.05
IL-1 β	1B	Control, TL2937, TL2766	<0.05
IL-6	1B	Control, TL2937, TL2766	N.S.
IFN- γ	1B	Control, TL2937, TL2766	N.S.
IL-10	1B	Control, TL2937, TL2766	<0.05
TGF- β	1B	Control, TL2937, TL2766	<0.05
MHC-II (CD11R1 high)	2	Control, TL2937, TL2766	<0.05
CD80/86 (CD11R1 high)	2	Control, TL2937, TL2766	<0.05
IL-10(CD11R1 high)	2	Control, TL2937, TL2766	<0.05
MHC-II (CD11R1 normal)	2	Control, TL2937, TL2766	<0.05
CD80/86 (CD11R1 normal)	2	Control, TL2937, TL2766	<0.05
IL-10 (CD11R1 normal)	2	Control, TL2937, TL2766	<0.05
MHC-II (CD11R1 low)	2	Control, TL2937, TL2766	N.S.
CD80/86 (CD11R1 low)	2	Control, TL2937, TL2766	N.S.
IL-10 (CD11R1 low)	2	Control, TL2937, TL2766	N.S.
IL-1 β	3A	ETEC control, TL2937+ETEC, TL2766+ETEC	<0.05
IL-8	3A	ETEC control, TL2937+ETEC, TL2766+ETEC	<0.05
IL-6	3A	ETEC control, TL2937+ETEC, TL2766+ETEC	<0.05
MCP-1	3A	ETEC control, TL2937+ETEC, TL2766+ETEC	<0.05
TGF- β	3A	ETEC control, TL2937+ETEC, TL2766+ETEC	<0.05
IL-1 β	3B	ETEC control, TL2937+ETEC, TL2766+ETEC	<0.05
IL-6	3B	ETEC control, TL2937+ETEC, TL2766+ETEC	N.S.
IFN- γ	3B	ETEC control, TL2937+ETEC, TL2766+ETEC	<0.05
IL-10	3B	ETEC control, TL2937+ETEC, TL2766+ETEC	<0.05
TGF- β	3B	ETEC control, TL2937+ETEC, TL2766+ETEC	N.S.
MHC-II (CD11R1 high)	4	ETEC control, TL2937+ETEC, TL2766+ETEC	<0.05
CD80/86 (CD11R1 high)	4	ETEC control, TL2937+ETEC, TL2766+ETEC	<0.05
IL-10(CD11R1 high)	4	ETEC control, TL2937+ETEC, TL2766+ETEC	<0.05
MHC-II (CD11R1 normal)	4	ETEC control, TL2937+ETEC, TL2766+ETEC	<0.05
CD80/86 (CD11R1 normal)	4	ETEC control, TL2937+ETEC, TL2766+ETEC	<0.05
IL-10 (CD11R1 normal)	4	ETEC control, TL2937+ETEC, TL2766+ETEC	<0.05
MHC-II (CD11R1 low)	4	ETEC control, TL2937+ETEC, TL2766+ETEC	<0.05
CD80/86 (CD11R1 low)	4	ETEC control, TL2937+ETEC, TL2766+ETEC	N.S.
IL-10 (CD11R1 low)	4	ETEC control, TL2937+ETEC, TL2766+ETEC	<0.05
MKP-1	5A	Control, TL2937, TL2766	<0.05
Bcl-3	5A	Control, TL2937, TL2766	<0.05
A20	5A	Control, TL2937, TL2766	<0.05
SIGIRR	5B	Control, TL2937, TL2766	N.S.
IRAK-M	5B	Control, TL2937, TL2766	N.S.
Bcl-3	5B	Control, TL2937, TL2766	<0.05
Body weight(24wk)	6	Control, Medium, TL2937, TL2766	<0.05
Plasma CRP	6	Control, Medium, TL2937, TL2766	<0.05
Plasma ACA	6	Control, Medium, TL2937, TL2766	N.S.
Carcass weight	7	Control, Medium, TL2937, TL2766	<0.05
Carcass backfat thickness	7	Control, Medium, TL2937, TL2766	<0.05
Unsaturated fatty acid	7	Control, Medium, TL2937, TL2766	N.S.
Tendernass	8	Control, Medium, TL2937, TL2766	<0.05
Juicy	8	Control, Medium, TL2937, TL2766	<0.05
Palatability	8	Control, Medium, TL2937, TL2766	<0.05
Plasma FFA	Suppl. Fig1	Control, Medium, TL2937, TL2766	N.S.
Plasma Glucose	Suppl. Fig1	Control, Medium, TL2937, TL2766	N.S.
Plasma TG	Suppl. Fig1	Control, Medium, TL2937, TL2766	N.S.
Plasma TC	Suppl. Fig2	Control, Medium, TL2937, TL2766	N.S.
Blood leucocytes	Suppl. Fig2	Control, Medium, TL2937, TL2766	N.S.
Granulocytes/Lymphocytes	Suppl. Fig2	Control, Medium, TL2937, TL2766	N.S.
Phagocytes activity	Suppl. Fig2	Control, Medium, TL2937, TL2766	N.S.
Blood antibodies	Suppl. Fig2	Control, Medium, TL2937, TL2766	N.S.
