# Supplementary material for: Immunobiotic Lactobacillus jensenii as immune-health promoting factor to improve growth performance and productivity in post-weaning pigs
Source: BMC Immunol. 2014 Jun 19;15:24. doi: 10.1186/1471-2172-15-24 (PMC4068960; doi:10.1186/1471-2172-15-24)
Supplement: Additional file 2: Figure S1 — Effect of Lactobacillus jensenii TL2937 on piglets’ plasma biochemical markers. Pigs were grown from 3 weeks of age until week 24. Five pigs were used for each experimental group. The Control group was fed only the balanced conventional diet without antimicrobials ad libitum. The Medium, TL2937 and TL2766 groups were fed balanced conventional diet with supplemental bacteria medium only (200 g/day), L. jensenni TL2937 (3 × 10^8^ cfu/g) or L. plantanum TL2766 (3 × 10^8^ cfu/g) respectively, from 3 to 17 weeks of age. Plasma levels of Free Fatty Acid (FFA), glucose, total cholesterol (TC) and triglycerides (TG) were determined at the end of experiments. Values for bars with different letters were significantly different (P < 0.05). Values for bars with shared letters do not differ significantly. [file 1471-2172-15-24-S2.pdf]

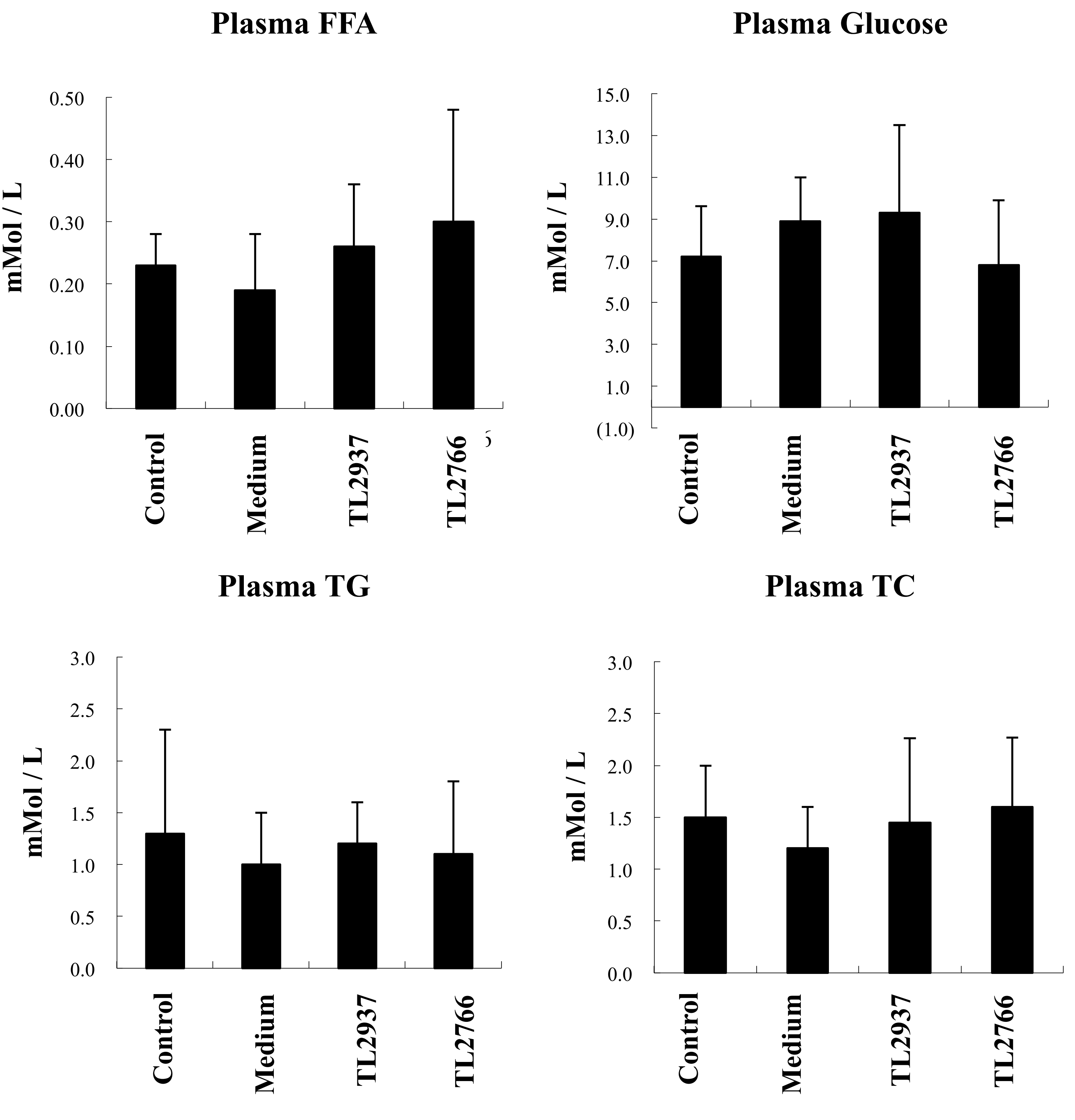

Supplemental Figure 1. Effect of *Lactobacillus jensenii* TL2937 on piglets' plasma biochemical markers. Pigs were grown from 3 weeks of age until week 24. Five pigs were used for each experimental group. The Control group was fed only the balanced conventional diet without antimicrobials *ad libitum*. The Medium, TL2937 and TL2766 groups were fed balanced conventional diet with supplemental bacteria medium only (200 g/day), *L. jensenni* TL2937 (3×10^8 cfu/g) or *L. plantanum* TL2766 (3×10^8 cfu/g) respectively, from 3 to 17 weeks of age. Plasma levels of Free Fatty Acid (FFA), glucose, total cholesterol (TC) and triglycerides (TG) were determined at the end of experiments. Values for bars with different letters were significantly different (P<0.05). Values for bars with shared letters do not differ significantly.
